# Selected Papers from the pHealth 2022 Conference, Oslo, Norway, 8–10 November 2022

**DOI:** 10.3390/jpm14090947

**Published:** 2024-09-06

**Authors:** Bernd Blobel

**Affiliations:** 1 Medical Faculty, University of Regensburg, 93053 Regensburg, Germany; bernd.blobel@klinik.uni-regensburg.de; 2 First Medical Faculty, Charles University Prague, 12800 Prague, Czech Republic; 3 Department of Informatics, Bioengineering, Robotics and System Engineering, University of Genoa, 16145 Genoa, Italy; 4 Faculty European Campus Rottal-Inn, Deggendorf Institute of Technology, 94469 Deggendorf, Germany

This Special Issue of the Journal of Personalized Medicine presents extended versions of selected contributions to pHealth 2022, the 19th International Conference on Wearable Micro and Nano Technologies for Personalized Health, held on 8–10 November 2022 in Oslo, Norway. The original papers were published in the IOS Press Studies in Health Technology and Informatics 2022, volume 299 (URL https://ebooks.iospress.nl/volume/phealth-2022-proceedings-of-the-19th-international-conference-on-wearable-micro-and-nano-technologies-for-personalized-health-810-november-2022-oslo-norway (accessed on 3 November 2022)).

The 2022 edition of pHealth continues to focus on the advancement of pHealth towards personalized, participative, preventive, predictive, and precision medicine (5P medicine), supported by technologies such as mobile technologies, micro/nano-bio smart systems, artificial intelligence and robotics, big data and analytics, and machine learning and deep learning. The new technologies offer new opportunities and bear new potential risks for security, privacy and safety. Therefore, ethical challenges and solutions for guaranteeing the needed trustworthiness must be addressed. Transformed health and social care ecosystems are highly distributed, complex and dynamic, combining perspectives and knowledge from different disciplines. This requires covering the medical, technological, political, administrative, and social domains, and philosophical or linguistic challenges in designing and managing such ecosystems. Bernd Blobel, the long-term Chair of the pHealth conference Scientific Program Committee as well as the pHealth Steering Committee, checked and edited every paper invited for publication in the MDPI *JPM* pHealth 2022 Special Issue before approving them for formal submission. Mauro Giacomini, as Co-Chair of both committees, managed the review process, which was performed by at least two independent international experts.

The book starts with challenges and solutions for healthcare transformation. In this chapter, a pHealth 2022 Keynote addresses principles and standards for designing and managing intelligent and ethically transformed health ecosystems [1]. After introducing the healthcare transformation towards 5P medicine, the paper explains the formal representation of health ecosystems as systems of systems, representing the different perspectives of the domains involved and all components, their functions and relationships at all granularity levels of interest, but also the viewpoints of the evolutionary or development process the ecosystem is facing. This must be carried out through a system-theoretical, architecture-centric, ontology-based, policy-driven approach, meanwhile standardized as ISO 23903 [2]. Interoperability and Integration Reference Architecture—Model and Framework. The Keynote paper explains the necessary principles and methodologies and references the standards defining them. The second contribution of the introductory chapter is a paper invited to the conference [3]. It observes the obstacles hindering capacity building and innovation promotion for eHealth in low- and medium-income countries exemplified for Africa. Here, the lack of financial resources, qualified personnel, but also infrastructure and governance must be mentioned.

The second chapter discusses and exemplifies the role of automation, machine learning and artificial intelligence for advancing pHealth. The first paper discusses the sharing of clinical data between healthcare establishments at a regional level in Italy, thereby guaranteeing the correct understanding of the exchanged data [4]. This requires the representational transformation of data and information shared using the Common Terminology Service Release 2 (CTS2) standard [5]. The second paper presents a methodology for improving the prediction of risks for COVID-19 infections depending on multiple factors such as environmental conditions and mobility of citizens, but also social factors [6]. For assessing the risk factors, a multidimensional analysis using machine learning models has been deployed, which is also applicable for other infectious diseases. As the best-performing model, the Extra Trees Regressor algorithm could be identified. The third paper in the chapter demonstrates the deployment of machine learning methodologies to identify and manage pregnancy and childbirth risks [7]. Thereby, data from clinical and laboratory tests and multiple measurements are deployed. The outcome could advance the decision support in perinatal care provision. The high predictive performance achieved by the models ensures precise support for both individual patient care and overall health organization management. The next paper of this chapter presents a new approach for evaluating human exercises in telehealth services to support remote diagnoses and treatment [8]. Thereby, the movement is defined as a static super object represented by a single two-dimensional image. This allows for analyzing and optimizing movements presented in videos. With proposed and demonstrated methodology, exercises can be simulated and scored. All papers discussed have been invited to the pHealth 2022 conference. The last paper, based on a presentation to pHealth 2021, investigates the improvement in the diagnosis and treatment of aortic stenosis (AS) by earlier detection using echocardiography and machine learning [9]. In this context, prevalence and clinical features of AS in patients with bicuspid aortic valves vs. patients with tricuspid aortic valve were studied. As an outcome, significant features impacting AS patients such as age, hypertension, aortic regurgitation, ascending aortic dilatation, and bicuspid aortic valves could be identified.

The third chapter is dedicated to security, privacy and safety as important prerequisites for the usability and acceptability of pHealth and dHealth solutions. The chapter starts with two papers invited to the pHealth 2022 conference. The first paper addresses one 5P medicine aspect, the individualization of the system according to the personal social, environmental, occupational and behavioral context of the subject of care, putting people at the center of the business system [10]. This is especially supported by Patient-Generated Health Data (PGHD). For understanding and correctly assessing opportunities and risks for the security, privacy and accuracy of PGHD, the actors involved, the technologies used, the specific types of data, their source and way of generation, etc., must be analyzed. Thereby, the Preferred Reporting Items for Systematic Reviews and Meta-Analyses (PRISMA) methodology was followed. The second paper presents a holistic view on privacy and trust, considering that pHealth ecosystems are multi-domain, highly distributed and dynamic, increasingly autonomous ecosystems deploying advanced technologies such as mobile and implantable sensors and actuators, big data and analytics as well as artificial intelligence [11]. Such systems enable the monitoring of the person’s physical and social life and behavior, making privacy and trust an illusion. Therefore, it is necessary to start the development of next-generation pHealth ecosystems according to ISO 23903 to formally and correctly present and manage intentions and objectives of the ecosystem actors as well as related consequences in each business use case. Thereby, personal health information (PHI) must be considered as personal property, and trust as a fiduciary duty, for the service provider and other stakeholders processing PHI in the ecosystem. The ecosystem’s behavior must be controlled by legally binding smart contracts stored in a blockchain-based repository. The last paper in this chapter deals with the assessment of potential risks of digital therapeutics (DTX) including health and wellness apps for patient safety, and possible adverse events [12]. By carrying this out, the authors developed a risk assessment canvas as a tool to calculate the risks bound to DTX. For defining relevant aspects, they used ISO/TS 82304-2 [13]. and performed a literature review.

The book concludes with a short eHealth solutions chapter, consisting of one paper on systems interoperability [14]. It demonstrates a practical solution for enabling the continuity of care. The challenges of continuity of care are the communication and cooperation between experts from different domains. For enabling this interoperability, a system of concepts to support continuity of care (ContSys) is inevitable. To formally represent ContSys, a ContSys ontology is necessary. For developing an IT solution, the business viewpoint components represented by the aforementioned ontology must be transformed into implementable artifacts. Here, HL7 Fast Health Interoperability Resources (FHIRs) are used.

The editors thank all authors and reviewers for their important contribution to the success of this volume. Furthermore, they are deeply indebted to the MDPI *Journal of Personalized Medicine* and its Editorial Office, especially to Penny Su, but also to Joanna Krefft, Marilyn Zhang, Calla Zhu and Jane Jin, for their continuous support. Without such efforts, this volume would not have been possible.
